# Kinetics of motile solitons in nematic liquid crystals

**DOI:** 10.1038/s41467-020-16864-8

**Published:** 2020-06-26

**Authors:** Satoshi Aya, Fumito Araoka

**Affiliations:** 1 South China Advanced Institute for Soft Matter Science and Technology (AISMST), School of Molecular Science and Engineering, South China University of Technology, Guangzhou, People’s Republic of China; 2 Physicochemical Soft Matter Research Team, RIKEN Center for Emergent Matter Science (CEMS), 2-1 Hirosawa, Wako, Saitama 351-0198 Japan

**Keywords:** Liquid crystals, Fluid dynamics

## Abstract

The generation of spatially localized, soliton-like hydrodynamic disturbances in microscale fluidic systems is an intriguing challenge. Herein, we introduce nonequilibrium solitons in nematic liquid crystals stimulated by an electric field. These dynamic solitons are robust as long as the electric field is maintained. Interestingly, their kinetic behaviours depend on the field condition—Tuning of the amplitude and frequency of the applied electric field alters the solitons to self-assemble into lattice ordering like physical particles or to command them to various dynamic states. Our key property to the realisation is the electrohydrodynamic instability due to the coupling between the fluid elasticity and the background convection. This paper describes a new mechanism for realising dynamic solitons in fluid systems on the basis of the electrohydrodynamic phenomena.

## Abstract (web summary)

Electric field induced convective instabilities in liquid crystal slabs can assume a localized shape. The authors show how to generate, manipulate and shepard these dissipative solitary excitations that do not require topological constraints for stabilization.

## Introduction

Solitons are conventionally known as spatially localised, shape-preserving travelling wave packets. Since the first observations of the solitonic behaviour of water waves by John S. Russell in 1834 and the introduction of the term of ‘soliton’ by Zabusky and Kruskal^1^, solitons have been identified in various natural phenomena. In theoretical frameworks such as the Korteweg–de Vries (KdV) or the nonlinear Schrödinger equation^2^, solitons appear as particle-like wave packets that travel without shape change and dissipation by balancing dispersive and nonlinear effects. This ideal behaviour of the solitons would enable the distortion-free long-distance transport of waves or structures, which has attracted considerable interest from both fundamental and technological viewpoints in many branches of science, including atmospheric circulation^3,4^, fluid^5^ and mechanical waves^6–8^, living organisms^9,10^, Bose–Einstein condensates^11–13^, light confinement, and propagation^14–16^.

Very recently, there have been several groundbreaking studies observing solitonic analogues in polar materials, such as spin systems^17–19^, ferromagnetic fluids^20^, and liquid crystals^21–26^. In most cases, these solitons possess topological singularities in spin-like vector fields that are distinct from the far-field background. In other words, the structures of the solitons are topologically protected. Mochizuki demonstrated a unidirectional thermally driven ratchet motion of magnetic skyrmions in the presence of a temperature gradient^17^. Smalyukh et al. experimentally and theoretically revealed that the twisted orientational order of chiral nematic liquid crystals under confinement induces assorted topological solitonic structures that can squirm in a specific direction under an electric field^20–22^. These topologically protected structures are static in thermal equilibrium but motile under an inhomogeneous electric field. Of course, since there are lots of examples of static topological structures known in many physical systems, these experimental facts do not guarantee all topologically protected structures to become motile. On the other hand, there have been reported intrinsically dynamic non-topologically protected solitons, where the KdV theory is applicable. As an example of such nontrivial solitons, Li et al. have recently observed travelling solitonic waves, which proposes a novel formation mechanism based on the flexoelectric effect in a non-chiral nematic liquid crystal^23,25,26^. While these previous works, including the ones published by Li et al.^25^ and Sohn et al.^26^ during the submission of the present paper, considered the mechanisms mainly based on the localised solitons without the electrohydrodynamic instability, still it is well known that electrohydrodynamic instability plays crucial roles in the formation mechanisms and dynamic behaviours of localised structures and patterns in many nonequilibrium fluid systems^27–29^. Therefore, employing the electrohydrodynamics is a fascinating strategy for exploration of dynamic solitons.

In this article, we show how solitons can arise in a parameter regime where the Carr–Helfrich electrohydrodynamic instabilities would be expected to occur. We even tune the anisotropies of both the dielectricity and the conductivity to generate a smooth path from standard flexoelectric or electrohydrodynamic states to dynamic solitonic states. The localised non-equilibrium excitation assumes the character of quasi-particles and enables various kinetics, e.g. directional translation, collision, reflection, and proliferation. The study establishes the fundamental physics and strategy of manipulating the kinetics of solitons in more general experimental settings and permits the observation of their time evolution, facilitating further experimental and theoretical studies of dynamic soliton excitations in soft-matter systems.

## Results

### Soliton induction

**Fig. 1:**
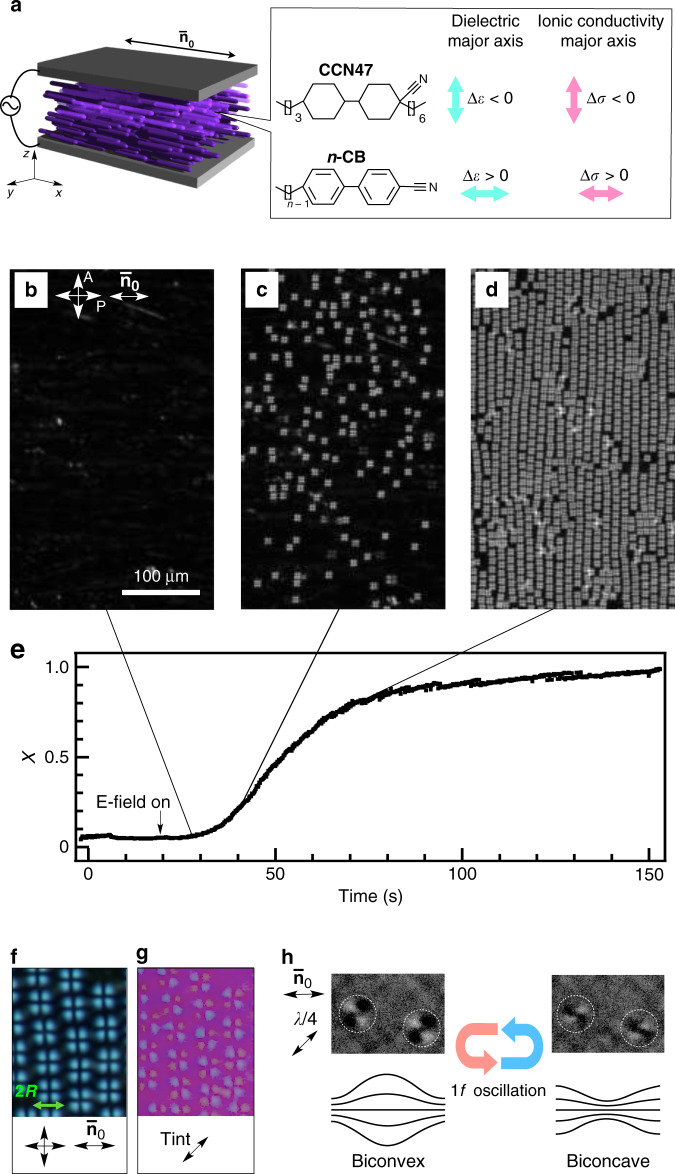
Creation of ordered solitons. **a** Slab geometry and chemical structures of the main materials—cyanobiphenyl (CB) and 4′-butyl-4-heptyl-bicyclohexyl-4-carbonitrile (CCN47). **b**–**d** Nucleation and growth processes of electrically pumped solitons visualised using polarising microscopy with crossed polarisers for specific times indicated by the time evolution of the soliton space-filling ratio, *X*, in **e**. Scale bar, 100 µm. The scale bar shown in **b** applies also to **c**, **d**. **e** Time evolution of the space-filling ratio of solitons. **f** Polarising microscopic images of the packed solitons. The soliton radius, *R*, is defined as the radius of the primary area undergoing elastic deformation. The space-filling ratio *X* is defined as the ratio of the total area of *N* solitons to the sample area, *A*. Image width, 65 µm. **g** Counterpart to **f** taken with a tint plate. **h** Monochromatic textures with a *λ*/4 plate and schematics of the alternating director field between two orientational states, taken with a high-frame-rate camera. Image width, 30 µm.

In contrast to refs. ^23,25^, we consider more general conditions accompanied by electrohydrodynamics, where dielectricity and ionic conductivity play important roles. In order to systematically examine the effects of the material parameters, we use the frustrative mixtures comprising two nematics, E7 (a mixture of cyanobiphenyl and cyanoterphenyl components) and 4ʹ-butyl-4-heptyl-bicyclohexyl-4-carbonitrile (CCN47) (Fig. 1a) from the beginning. By altering the concentration of E7 or adding excesses of the ionic species tetrabutylammonium benzoate (TBABE) to the CCN47, we can facilitate a continuous transition of the anisotropic dielectric/conductivity states from (−−) to (++), where the pairs of the signs, (Δ*ε*Δ*σ*) (the so-called de Gennes’ notation^28^), are given corresponding to the signs of the dielectric and conductivity anisotropies, ${\mathrm{\Delta }}\varepsilon = \varepsilon _\parallel - \varepsilon _ \bot$ and ${\mathrm{\Delta }}\sigma = \sigma _\parallel - \sigma _ \bot$. The subscripts || and $\bot$ indicate the directional components parallel and perpendicular to the average molecular orientation, denoted by the nematic director ${\bar{\mathbf{n}}}$, respectively. In particular, we pass the (−+) state, characteristic for the electrohydrodynamic instability of the Carr–Helfrich phenomenology.

**Fig. 2:**
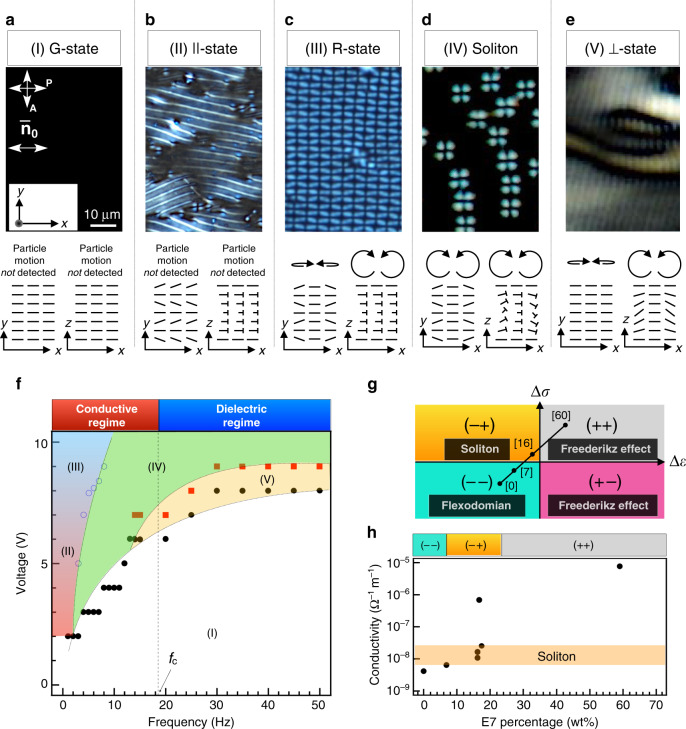
Conditions for generating solitons. **a**–**e** Polarising microscopic textures of the orientational states and the schematics of the director field by nail notation and motion trajectories of particle in the presence of an electric field: **a** 2 V, 10 Hz; **b** 3 V, 2 Hz; **c** 8 V, 2 Hz; **d** 6 V, 10 Hz; **e** 7 V, 23 Hz. The director field in the *xy* plane is on the middle plane of the cell. Scale bar, 10 µm. The scale bar shown in **a** applies also to **b**–**e**. **f** State diagram as a function of the voltage and frequency. The markers mean the separating boundaries of the different states. The dot lines are drawn to increase the visibility of the state diagram. All the transitions between different states exhibit coexistence of neighbouring states (see Supplementary Fig. 6). **g** Dominating phenomena occurring because of the changes in the signs of the anisotropies of dielectricity (Δ*ε*) and conductivity (Δ*σ*), observed in the range from 3 to 10 V, at frequencies <20 Hz. The solid line indicates a route for tuning the combination of anisotropies by mixing E7 at different weight percentages, which are shown in square brackets, with CCN47. **h** Change in the specimen conductivity obtained by changing the concentration of E7 in CCN47.

Through extensive experiments, we find that solitons can be created in the frequency range of 2–60 Hz and for mixtures of E7–CCN47 with 15–18 wt% E7 (see also the state diagrams in Fig. 2f–h). As seen in the generalised scheme of the procedural conditions for the solitons (Fig. 2g), stable solitons are observed only in (−+) at low electric fields (≤10 V), that is, a typical condition in which the Carr–Helfrich electrohydrodynamics becomes active^27–29^. In this case, a convective flow occurs parallel to the background nematic director ${\bar{\mathbf{n}}}$, while in contrast, no observable flow occurs for (−−) and (++) accompanied with the flexoelectric effect and Freederikz effect-driven uniform reorientation, respectively. The present mixture system does not exhibit (+−), but it is known in the conventional systems that the Freederikz effect dominates in (+−).

As expected, conductivity is vital in determining the stability of the solitons in the present system, i.e. the solitons appear in the limited range of the moderate conductivity, $8\,\times\,10^{ - 9}\,<\,\sigma\,<\,4\,\times\,10^{ - 8}\,{\mathrm{\Omega }}^{ - 1}\,{\mathrm{m}}^{ - 1}$ (Fig. 2h). However, this conductivity value is much lower than those required for the conventional Carr–Helfrich electrohydrodynamic phenomena. For example, even in the (+−) state of almost the same E7–CCN47 mixture with approximately 16 wt% E7, there emerges only the global flow pattern without isolated solitons if too many ions are added and the conductivity exceeds the above-mentioned range. This means, the conventional convection regime upon the Carr–Helfrich electrohydrodynamic effect occurs everywhere in the material. On the other hand, in the case of too few ions and hence too low conductivity, the electrohydrodynamic effect is suppressed and the Freederikz effect dominates over the pattern formation (see Supplementary Note 11). The similar low-conductivity condition has been tested by Li et al. for the (−−) state, where the flexoelectric effect works instead of both the Carr–Helfrich electrohydrodynamic and the Freederikz effects^23^. One possible physical situation deduced from these facts is that some localised effect on the top of the Carr–Helfrich electrohydrodynamic mechanism is happening for the emergence of the present solitons: the solitons are a superposition of the electrohydrodynamic rolls, which can coexist with the global electrohydrodynamic roll patterns (states (II), (III), and (V)) as discussed later (see also Supplementary Note 8 and Supplementary Movie 12). Indeed, as evidenced in Supplementary Notes 1 and 2, and Supplementary Figs. 1 and 2, the ions are considered to be localised to produce independent solitons, compared to the relatively uniform spatial distribution of the ions for the global flow pattern based on the conventional Carr–Helfrich electrohydrodynamics. In such ion-rich areas, the electrohydrodynamic flow can easily occur due to the larger hydrodynamic torque^29^.

In Fig. 1b–d and Supplementary Movie 1, we show time-dependent microscopic texture images acquired during soliton creation in a mixture containing 16 wt% E7 for a 10 V electric field at 12 Hz at the specific times designated in Fig. 1e. Independent cruciform solitons are randomly excited in space and time (Fig. 1b, c), while they disappear when the field is absent (Supplementary Fig. 1). These solitons fluctuate around and remain relatively near their induction positions with a space-filling ratio of *X* = *πR*^2^/*A* < 0.6, where *R* and *A* are the soliton radius and the total area of the region, respectively. Since the time dependence of *X* reflects how the involved ions localise, it is fitted by the Kolmogorov–Johnson–Mehl–Avrami expression, which might give insights into the procedure of the migration of the ions during the soliton generation (Supplementary Notes 1 and 2, Supplementary Figs. 1–3), $\log \left[ {\log \left[ {\left( {1 - X} \right)^{ - 1}} \right]} \right] = m\log t + \log K$, where *X*, *t*, *K*, and *m* are the space occupancy of the director-deformed region, the time, the temperature-dependent Avrami coefficient, and the exponent, respectively^30–32^. The value obtained for *m* is 2.9, suggesting the involvement of two-dimensional homogeneous nucleation and growth processes^33^ triggered by the localisation of ions.

### Structure of solitons

Centred rectangular packing of the solitons occurs when the filling ratio *X* > 0.6 (Fig. 1d, Supplementary Fig. 4), a value consistent with the close-packed ratio of perfectly hexagonally packed solid spheres in two dimensions (*X* = 0.604). For such packing states, the solitons are fusion resistant. Similar packing structures occur in some self-driven systems^23,25,34,35^. Next, we investigate the director structure of each soliton. Under an exposure time sufficiently longer than the oscillatory period of the applied electric field, the dynamic structural properties of the solitons cannot be captured and only a time-averaged structure is clarified. Figure 1f, g show magnified views of the time-averaged solitonic structures obtained with an exposure time of 200 ms under crossed polarisers and with a standard tint waveplate (retardation equal to 530 nm) taken by a conventional camera. An array of the concentric patterns of the time-averaged solitons pretends as if *s* = +1 topological defects ordering in the midplane of the nematic film. In order to elucidate the dynamic structure, we perform high-frame-rate polarising microscopy at 100 frames per second. Figure 1h shows a time-dependent oscillation of the structure of the solitons. It becomes clear that the director field of the solitons transforms between two defectless structures, i.e. biconvex and biconcave structures, synchronised with the applied electric field. It is noted that, by analysing the director fields, the biconvex structure has a larger deviation angle of the director in the midplane of the nematic film than the biconcave structure (time-resolved structures in Supplementary Fig. 5). This explains why the time-averaged structure looks like a concentric domain with a *s* = +1 defect.

As the soliton-filling ratio increases, mutual repulsion, separating the solitons by distances of about twice the soliton radius, is observed (Supplementary Movie 1). As a result of this repulsion, the solitons become resistant to the external stress without coalescence, confirming the pseudoparticle nature of the solitons.

### Conditions of soliton creation

The orientational pattern that includes the solitons varies depending on the voltage and frequency (Fig. 2a–e). On the basis of the director deformations and flow profiles, we classify the frustrated states into five categories: (I) ground planar alignment (G-state), (II) ||-roll pattern (||-state), (III) rectangular pattern (R-state), (IV) soliton state (Soliton-state), and (V) $\bot$-roll pattern ($\bot$-state). Figure 2f shows a state diagram for a mixture containing 16 wt% E7, which exhibits (−+) anisotropy.

In the G-state, neither elastic deformation nor hydrodynamic effect occurs; therefore, the initial uniform uniaxial alignment is maintained. In the ||-state, which appears in the lower-frequency region <9 Hz, only an in-plane splay elastic deformation of the director occurs because of the coupling with the flexion-induced polarisation, the so-called flexoelectric effect^27,36^ Because the strong surface-anchoring effect forces fix the surface directors in the initial state, twist deformation along the electric field direction (*z* direction) is induced, yielding a one-dimensional periodic pattern. Particle tracking confirms the absence of flow (see ‘Methods’). An increase in the voltage in the ||-state causes a field-induced hydrodynamic convective flow parallel to the initial director (${\bar{\mathbf{n}}}_0$). The coupling between the flow and the flexoelectric effect induces a two-dimensional version of the ||-state, i.e. the R-state. The $\bot$-state occurs in a dielectric regime in which ions are effectively immobile, but the nematic director oscillates with the applied electric field. The convective flow field parallel to ${\bar{\mathbf{n}}}_0$ arising from an electrohydrodynamic effect (Carr–Helfrich instability) yields a stripe pattern in which the director has a wavy modulation in space, as observed in nematics with (−+) anisotropy^29^. The appearances of the respective roll patterns in the ||-state and $\bot$-state significantly differ in terms of the roll direction: rolls in the ||-state run parallel to the director; those in the $\bot$-state are perpendicular. The Soliton-state occurs in the frequency range between the ||-/R-state and $\bot$-state and spans a conductive regime in which ionic convection dominates the dielectric oscillation of the director. Accordingly, the soliton system has features of both the ||-state and $\bot$-state with a compresence of elastic and convective effects, as discussed below. The way the solitons are observed to move in our system depends on frequency as well as on the amplitude of the voltage applied. For instance, at constant frequency but with the increased voltage of the electric field (and thus the director oscillation), a transition from a motional pattern coined ‘swimming’ to ‘proliferation’ is observed. Worth noting that voltage or frequency contributes differently to the kinetics of the solitons as discussed below.

### Collision and elastic reflection in the swimming regime

**Fig. 3:**
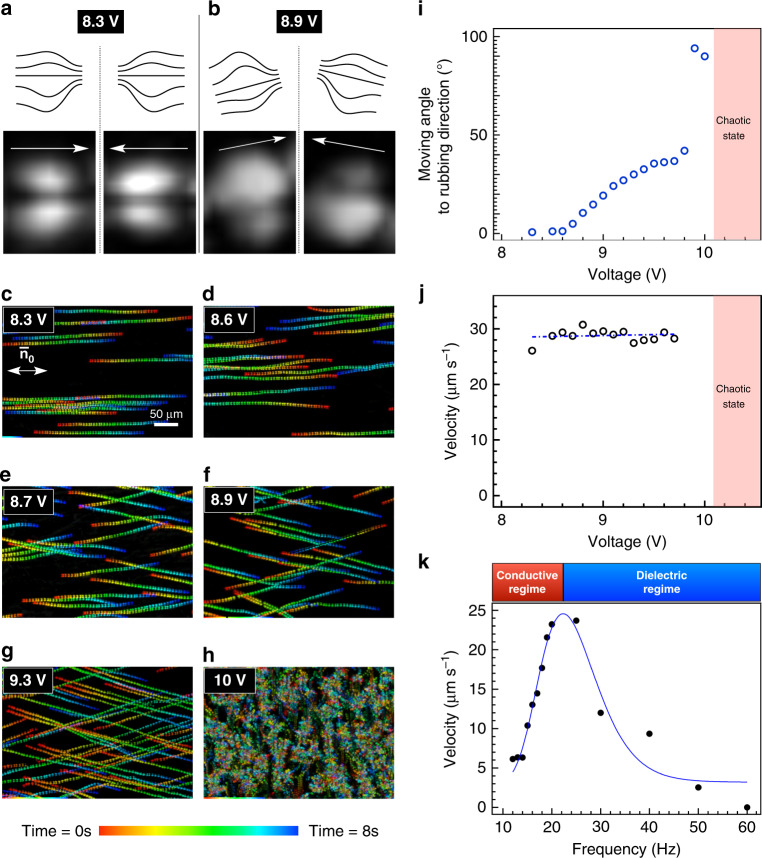
Time-dependent dynamics versus electric-field parameters. **a**, **b** Time-averaged director distortion of the kinetic solitons at 8.3 and 8.9 V. Schematics of the alternating distorted convex and distorted concave director fields are presented with magnified patterns of them. Image width, 11 µm. **c**–**h** Typical time-dependent trajectories of solitons during different motions at various voltages at 20 Hz, stacked over 8 s. Scale bar, 50 µm. The scale bar shown in **c** applies also to **d**–**h**. **i** Angles of soliton motion with respect to ${\bar{\mathbf{n}}}_0$ at 20 Hz. **j** Velocity of the solitons at 20 Hz plotted as a function of the voltage. **k** Velocity of solitons at 10 V as a function of the frequency.

An increase in the voltage or frequency in the oscillation regime (the transition from the orange to purple-blue regions in Supplementary Fig. 7) produces steady directional motion of the solitons in which the initial time-averaged cruciform configuration changes to a time-averaged bug-eye-like pattern because of asymmetric elastic deformation (Fig. 3a, b). In this regime, the director structure of the solitons during the motion is also dynamically oscillating with time (Supplementary Fig. 8). The direction of the motion can be tuned by changing either the amplitude or frequency of the electric field. Figure 3c–h show the time-dependent trajectories of solitons under the control of the tuned amplitude of the electric field at a characteristic frequency of 20 Hz, where the solitons are most stable in their shape. At low voltages (8.2 < *V* < 8.5 V, Fig. 3c), the solitons move directionally parallel to ${\bar{\mathbf{n}}}_0$ in the *xy* plane. Notably, they randomly move to the left or right because of the previously discussed dielectric oscillation. As plotted in Fig. 3i, at *V* > 8.5 V, the directional angle of motion with respect to ${\bar{\mathbf{n}}}_0$ continuously varies as the voltage is increased (Fig. 3d–h, Supplementary Movies 3–8), corresponding to the increasingly asymmetric deformation of the bug-eye-like pattern (Fig. 3b). High-speed camera observations reveal that this movement is driven by the oscillation of the director field between distorted versions of two orientational states, as shown in Fig. 1h (Supplementary Movie 9). The directional motion ends at about 10 V, after which the soliton motion becomes somewhat chaotic, leading to a fractalised proliferation process that will be discussed later. The average velocity of the motion of the solitons is measured through a trajectory tracking as shown in Fig. 3c–h and plotted as a function of voltage and frequency in Fig. 3j, k. Although the velocity is nearly constant with respect to the voltage because the background flow field is mostly unaffected in the limited voltage range, a significant frequency dependence is observed. The solitons are resonantly active at about 20 Hz, as shown in Fig. 3k. This frequency corresponds to a critical frequency that separates the conductive and dielectric regimes^29^, as described by the competition between the relaxation process of mobile charge in space and the dielectric relaxation of the director,

1 $$f_{\mathrm{c}} = \frac{{\sigma _ \bot }}{{\varepsilon _0\varepsilon _ \bot }}.$$

Given *σ*_⊥_ = 10^−9^ Ω^−1^ m^−1^ and $\varepsilon _ \bot$ = 4, as measured experimentally, we obtain the critical frequency of 28 Hz by calculation, consistent with the experimentally observed value.

**Fig. 4:**
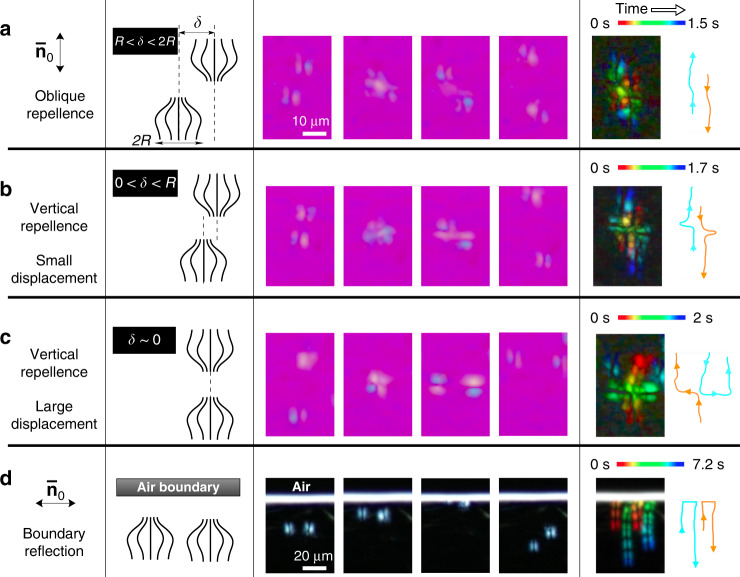
Elastic collisional trajectory tracking. **a**–**c** Snapshots of collisions between solitons (with a tint plate) and **d** reflection of solitons by an air boundary (with crossed polarisers) with schematics of solitonic topologies. In the second column from the left, the schematics of time-averaged distorted biconvex director fields before collisions are drawn. Instantaneously, the director field alternates between distorted versions of two orientational state, as shown in Fig. 3a. Dashed lines in the schematics are used to clarify the positional offset, *δ*. The overall trajectories are shown with colour-code traces and arrows in the right-most column. Scale bar, 10 µm, in (**a**–**c**). Scale bar, 20 µm, in (**d**).

The dynamics of the collisions between solitons and their reflection by obstacles can be understood in terms of their pseudoparticle nature. Figure 4a–c and Supplementary Movie 10 show that their head-on collisional behaviour varies depending on the degree of offset, *δ*, between soliton cores. When *R* < *δ* < 2*R*, interference occurs between the peripheries of soliton pairs with the same orientation. Consequently, the solitons repel each other at a small post-collision angle; at 0 < *δ* < *R*, elastic deformation becomes more significant because of the increased mismatch of the director field between solitons, leading to vertical repulsion between the solitons. Following this collision, the solitons separate such that elastic deformation is avoided and then continue to move along their original directions of motion. Interestingly, the solitons are elastically reflected at an air–nematic interface in the manner of real particles colliding with a wall (Fig. 4d).

### Proliferation regime

**Fig. 5:**
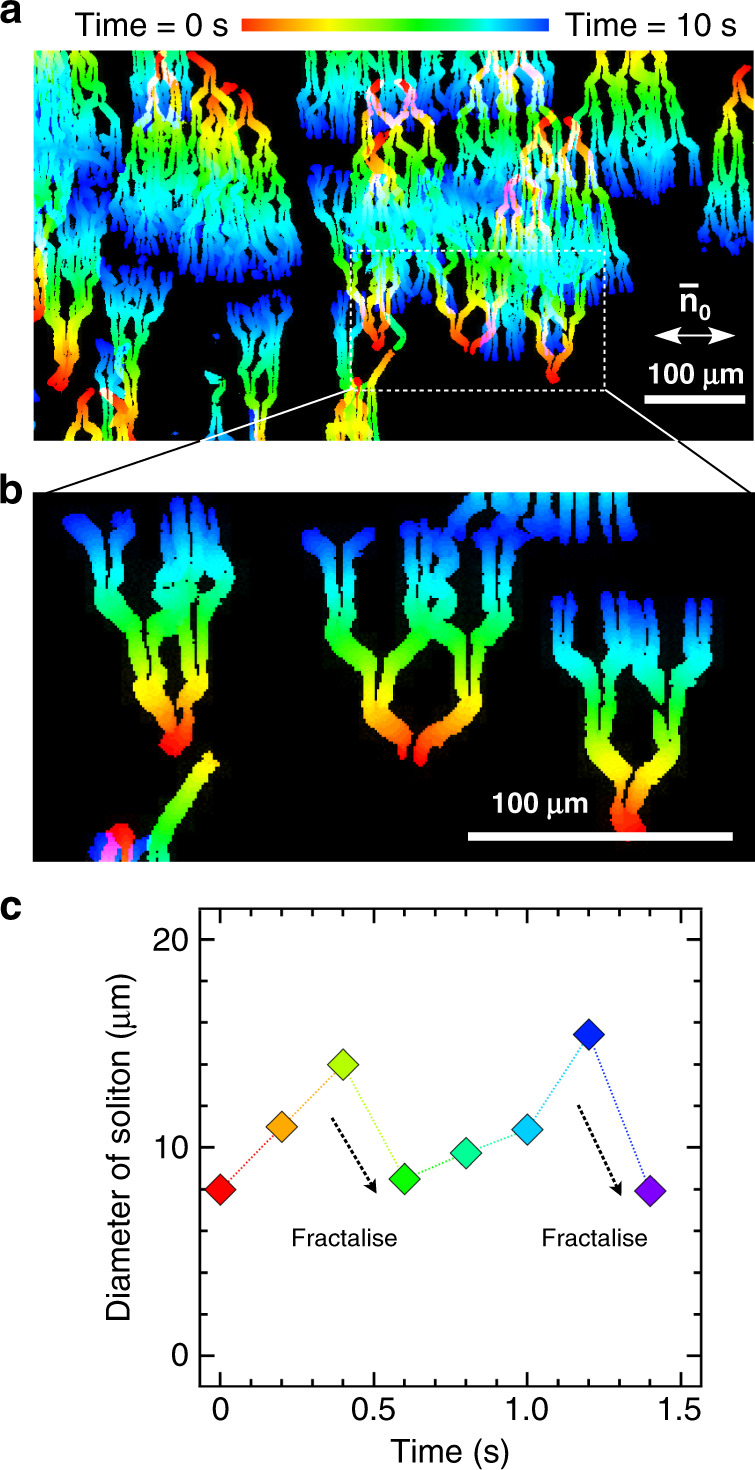
Fractal cloning of solitons. **a** Time-dependent trajectories of solitons during fractal cloning stacked over **a** 10 s and **b** 5 s. Scale bars, 100 µm. **c** Fractalisation occurring when the soliton size is nearly doubled.

Figure 5a, b show the fractalised proliferation trajectory of destabilised solitons for a 10 V field at 20 Hz (Supplementary Movie 11). Soliton fractalisation produces clones for movement perpendicular to ${\bar{\mathbf{n}}}_0$. Upon fractalisation, the solitons bifurcate and move obliquely for a short distance. Their dynamic structure is preserved during this process, and the emerged two solitons have an opposite phase in their orientational structure. The fractalisation process corresponds to the continuous growth in soliton size. Considering that an optimum size exists for the solitons at a fixed film thickness (Supplementary Fig. 11), the fractalisation process results in the continuous accumulation of excess elastic energy. The solitons fractalise when the energy penalty from elastic deformation cannot be compensated by the effects of surface anchoring and dielectric interaction. This is illustrated in Fig. 5c, which shows the soliton diameter, 2*R*, as a function of time; fractalisation occurs when the soliton size exceeds a specific value. Notably, the fractalisation simultaneously occurs for all solitons (Fig. 5a, b). This implies that a synchronised growth mechanism is involved for the solitons, resulting in a solitonic trajectory with a Hausdorff fractal dimension of *D* = 1.

## Discussion

The localised solitary oscillating director structures can also exist under conditions for global flow patterns to occur; there is a coexistence of states (IV), Soliton-state, and (V), the $\bot$-state (see Fig. 2). The involved hydrodynamic flow can be used to push the soliton in a certain direction. In the Soliton-state, convective flows with waves obliquely travelling to both the left and right relative to ${\bar{\mathbf{n}}}_0$ simultaneously appear, as confirmed by the observation of roll patterns coexisting with solitons in the $\bot$-state (Supplementary Movie 12). The localised dynamics are assumed to consist of two oblique waves travelling at an angle *θ* with respect to ${\bar{\mathbf{n}}}_0$, as introduced elsewhere^37–39^. As a first-order approximation, the amplitude expansion of the flow field is,

2 $$w = \left[ {A\left( {r,t} \right)e^{ik_{\mathrm{A}} \cdot r} + B\left( {r,t} \right)e^{ - ik_{\mathrm{B}} \cdot r}} \right]e^{\left( {ik_{\mathrm{C}} \cdot r - \omega t} \right)}\sin z,$$

where *A* and *B* are the respective spatiotemporal variable amplitudes of the two assumed travelling waves; *k*_*A*_, *k*_*B*_, and *k*_*C*_ are the wavenumbers; *r* is the spatial coordinate; *t* is time; and *ω* is the angular frequency. For small amplitudes $A\left( { = A_0e^{ - i\left( {Qx + Py - \Omega t} \right)}} \right)$ and $B\left( { = B_0e^{ - i\left( {Qx - Py - \Omega t} \right)}} \right)$, where Ω is the angular frequency, the description of the flow waves in the Ginzburg–Landau framework is valid, yielding

3 $$\frac{\partial }{{\partial t}}A = \frac{\partial }{{\partial A}}\left[ { - {\int} {{\mathrm{d}}V\left( {{\mathbf{u}}_A \cdot \nabla A} \right) + \left( {\mu - c\left| A \right|^2 - g\left| B \right|^2} \right)A^2 + \left( {\nabla A} \right)^2} } \right]$$

and

4 $$\frac{\partial }{{\partial t}}B = \frac{\partial }{{\partial B}}\left[ { - {\int} {{\mathrm{d}}V\left( {{\mathbf{u}}_B \cdot \nabla B} \right) + \left( {\mu - c\left| B \right|^2 - g\left| A \right|^2} \right)B^2 + \left( {\nabla B} \right)^2} } \right],$$

where **u**_A,B_ are the group velocities of the waves, *μ* is the bifurcation parameter, and *c* and *g* are coupling coefficients. Forming a symmetric superposition mode of the two waves in the *xy* plane with a finite Ω proportional to $\left| {A_0} \right|^2$ produces a two-dimensional localised travelling rectangle solution corresponding to a solitonic state. If only the flow field is under action, two types of solitonic motion are possible: a directional motion parallel to ${\bar{\mathbf{n}}}_0$ and a two-dimensional random walk within the *xy* plane. However, in our results, variable angles of solitonic motion with respect to ${\bar{\mathbf{n}}}_0$ are achieved. This motivates us to consider the elastic deformation of the bulk directors by introducing a spatially modulated imbalance of amplitudes between *A* and *B*. Although convective flow is considered in ref. ^37^, our solitons have splay deformation in the *xy* plane and twist-bend deformation in the *xz* plane (Fig. 2d) in addition to a convective flow. Owing to the flow-alignment property of the director, its orientation tends to be parallel to the flow field. Since the orientation of the director oscillates with time, the local flow also changes its direction accordingly through a self-regulating procedure (Supplementary Fig. 12). Such processes can trigger the local motion of topological defects^40^, thereby providing an additional travelling mode out of the *xz* plane. Indeed, the electrohydrodynamic convection flow can be modulated by twist deformation to produce a spiral flow^41^. Because the elastic deformation in the *xy* plane weakens in the transition from the ||-state through the Soliton-state to the $\bot$-roll state as the frequency is increased or the voltage is decreased, the inclination of the oblique solitonic movement decreases with respect to ${\bar{\mathbf{n}}}_0$; with neither flow nor elastic deformation in the *xy* plane, no two-dimensional motion is observed in the studied parameters (Fig. 2a–e). Thus the local coupling between the elastic deformation and the flow field in an anisotropic medium permits effective dictation of the solitonic motion directionality, which cannot be achieved through a flow field alone.

**Fig. 6:**
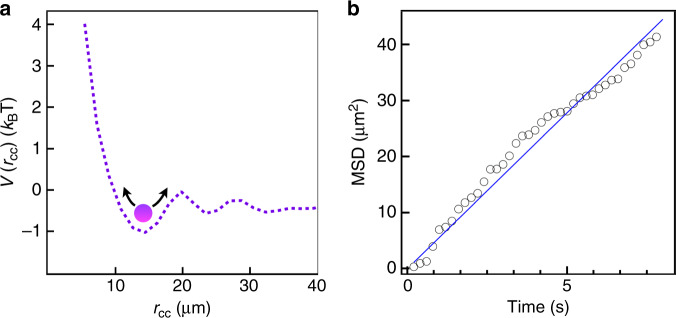
Pseudoparticle properties of solitons. **a** Pairwise soliton potential. **b** MSD of solitons as a function of time.

Next, we discuss the pseudoparticle properties of solitons in anisotropic flows. For characterisation, we analyse a radial distribution function of packed solitons, *g*(*r*_cc_) (Supplementary Fig. 13) and the thermal energy-activated solitonic Brownian diffusion. Figure 6a shows the pairwise potential *V*(*r*_cc_) as a function of the centre-to-centre distance between solitons, *r*_cc_. At the dilute limit, *V*(*r*_cc_) is directly calculated as $- k_{\mathrm{B}}T\log \left[ {g\left( {r_{{\mathrm{cc}}}} \right)} \right]$. The first potential closely corresponds to the mean nearest-neighbour distance between solitons, *r*_cc,o_ = 14.2 µm. The depth of the potential well slightly exceeds the thermal energy, *k*_B_*T*, which is consistent with the observed minimal soliton fluctuation beyond *r*_cc,o_. The solitonic Brownian diffusion process is calculated as the two-dimensional mean square displacement of the solitons over time *t* (Fig. 6b). The effective diffusion constant of the solitons is 1.4 µm^2^ s^−1^, corresponding to a mean solitonic fluctuation velocity of about 1 µm s^−1^. The effective mass of the solitons, *m*_eff_, is expressed as

5 $$U = \frac{1}{2}m_{{\mathrm{eff}}}v^2,$$

where *U* is the kinetic energy and *v* the mean velocity of the solitonic fluctuation. By balancing the thermal energy, *k*_B_*T*, and the kinetic energy of the solitons, *m*_eff_ is estimated to be 1 picogram. In contrast, the real mass of molecules constituting each soliton is estimated to be *ρπR*^2^*h* ≈ 1000 picograms, where *ρ* and *h* are the material density and nematic film thickness, respectively. The effective mass of the soliton is significantly smaller than the real mass of the materials constituting the solitons. Notably, *m*_eff_ is independent of the film thickness irrespective of the change in the real mass (Supplementary Fig. 14). Since the present soliton is a localised deformation of the director field as shown in Fig. 2d, such a time-averaged three-dimensional state is approximated using a spherical distortion,

6 $$\theta \left( {x,y,z} \right) \propto \sin \left[ {\frac{{\pi x}}{R}} \right]\cos \left[ {\frac{{\pi y}}{R}} \right]\cos \left[ {\pi \left( {\frac{z}{d} - 0.5} \right)} \right],$$

where *R* and *d* are radius of the solitons and the film thickness with a relationship of *R* ∝ *d* as shown in Supplementary Fig. 11. The elastic energy of the solitons is calculated by considering the Frank elasticity:

7 $$F = \frac{1}{2}{\int} {\left[ {K_{11}\left( {\nabla \cdot {\mathbf{n}}} \right)^2 \,+\, K_{22}\left( {n \cdot \left( {\nabla \times {\mathbf{n}}} \right)} \right)^2 \,+\, K_{33}\left( {n \times \left( {\nabla \times {\mathbf{n}}} \right)} \right)^2} \right]{\mathrm{d}}V} ,$$

with different film thicknesses, by using experimentally determined splay (*K*_11_ = 13 pN), twist (*K*_22_ = 6.2 pN), and bend elastic constants (*K*_33_ = 15 pN). Therefore, the decrease of elastic energy density upon increasing the film thickness is compensated by the increase of the volume of the soliton, leading to the invariance of the total elastic energy (Supplementary Fig. 15). This implies that the constant *m*_eff_ reflects the elastic energy stored by the elastic deformation in the director field.

In summary, we have demonstrated the generation of nonequilibrium solitons and the control of their kinetics in nematic liquid crystals. Unlike the well-known Russell’s solitons that propagate in isolation after their excitation, the present nonequilibrium solitons are structurally robust upon application of an electric field and dissipate after the field is turned off. It is revealed that the stabilisation of the solitons is rooted in the so-called Carr–Helfrich electrohydrodynamic mechanism, where the interactions between background flow and elastic deformation play important roles. Interestingly, these solitons show unique dynamic behaviours, i.e. controllable directional swimming and proliferation. The results suggest a new possible pathway that solitons can be made and manipulated in nonequilibrium conditions based on the electrohydrodynamic mechanism in anisotropic fluid systems.

## Methods

### Sample preparation

The frustrative nematic mixtures were formed by mixing E7 (Wako, Aldrich, and made in house) with CCN47 (Nematel GmbH) by weight. These molecules form a uniaxial nematic phase in which the average molecular orientation is characterised by a vectorial director, ${\bar{\mathbf{n}}}$. Because of the macroscopic and nonpolar nature of the nematic phase, the head and tail of ${\bar{\mathbf{n}}}$ are equivalent. The liquid crystals are uniformly aligned between two glass plates with a controlled thickness in the range of 2–50 µm. In the manuscript, if not specified, we show solitonic behaviours in a 5.2-µm-thick film. Although E7 has positive anisotropies (Δ*ε*Δ*σ*) = (++), those of CCN47 are negative (Δ*ε*Δ*σ*) = (−−). TBABE was added to the E7–CCN47 mixture to tune its net strength of conductivity. Films comprising these mixtures were sandwiched between pairs of substrates coated with rubbed polyimide on indium-tin-oxide electrode layers, with the film thickness adjusted by adding silica particles to obtain a 2–12-µm-thick film (micromer, Micromod) or by adding polyester film spacers to obtain a 20–50-µm-thick film (Mylar, Dupont). To create solitons, a sinusoidal or rectangular AC electric field is applied normal to the plates (Fig. 1a). In many other liquid crystalline systems, including Schiff bases and cyanobiphenyl mesogens, solitons were also found.

### Conductivity measurement

The electrical and dielectric responses in the liquid crystal mixtures were characterised via dielectric spectroscopy (Solartron Analytical, impedance/gain-phase analyser 1260A with dielectric interface system 1296A). An AC electric field at 0.05 V_rms_ sweeping at a frequency of 10^−2^–10^4^ Hz was applied to the films, and the resulting impedance response was recorded and analysed (Supplementary Fig. 16).

### Director mapping

The orientation of the directors was determined optically using polarising microscopy. The highest birefringence of the mixtures was sufficiently low (0.03 < **Δ*n*** = ***n***_**e**_ − ***n***_**o**_ < 0.08, where ***n***_**e**_ and ***n***_o_ are the extraordinary and ordinary refractive indices, respectively) to carry out this task. On the basis of the measured transmittance of light through the films, we calculated the orientation of the directors. Our method was the same as that presented in ref. ^20^. We also conducted fluorescence confocal polarising microscopy (TCS SP8 STED, Leica) to probe the three-dimensional distribution of the soliton directors. Although volume scanning was performed during soliton movement, owing to their fast fluctuation that was synchronised with the electric field, the solitons were not clear enough to allow their analysis. Instead, we measured the emission intensities at single planes on the surfaces of the slab and in the middle of the cell. The intensity on the surfaces was nearly constant during the application of the electric field, confirming a strong surface-anchoring effect. The signal was maximum at the middle of the slab, corresponding to elastic deformation of nearly zero on the surface that reached a maximum in the middle of the slab.

### Characterisation of flow field

The particle tracking method was used to visualise the effective flow in the mixtures in the presence of an electric field. In this process, a small number of 2-µm particles were dispersed into the nematic mixtures, and their motions were tracked by both bright-field and polarising microscopy.

### Time-dependent trajectory analysis

The recorded dynamics of the solitons were analysed using custom-made ImageJ macros. For the precise image analysis needed to extract sharp shapes and calculate the centres of gravity of the solitons, a dark but slightly structured time-dependent background was averaged over time.

## Supplementary information

Supplementary Information

Description of Supplementary Files

Supplementary Movie 1

Supplementary Movie 2

Supplementary Movie 3

Supplementary Movie 4

Supplementary Movie 5

Supplementary Movie 6

Supplementary Movie 7

Supplementary Movie 8

Supplementary Movie 9

Supplementary Movie 10

Supplementary Movie 11

Supplementary Movie 12

## Data Availability

All data that support the findings in this study are available in the article and in Supplementary Materials. Additional information is available from the corresponding author upon reasonable request.
