# Supplementary material for: Kinetics of motile solitons in nematic liquid crystals
Source: Nat Commun. 2020 Jun 26;11:3248. doi: 10.1038/s41467-020-16864-8 (PMC7319993; doi:10.1038/s41467-020-16864-8)
Supplement: Supplementary file 1 — Supplementary Information [file 41467_2020_16864_MOESM1_ESM.pdf]

Supplementary Information

Kinetics of motile solitons in nematic liquid crystals

Satoshi Aya[1*] and Fumito Araoka[2*]

[1] *South China Advanced Institute for Soft Matter Science and Technology (AISMST), School of Molecular Science and Engineering, South China University of Technology, Guangzhou, People's Republic of China*

[2] *Physicochemical Soft Matter Research Team, RIKEN Center for Emergent Matter Science (CEMS), 2-1 Hirosawa, Wako, Saitama 351-0198, Japan*

[*] Corresponding Authors: S.A. (satoshiaya@scut.edu.cn) and F.A. (fumito.araoka@riken.jp)

Supplementary Information

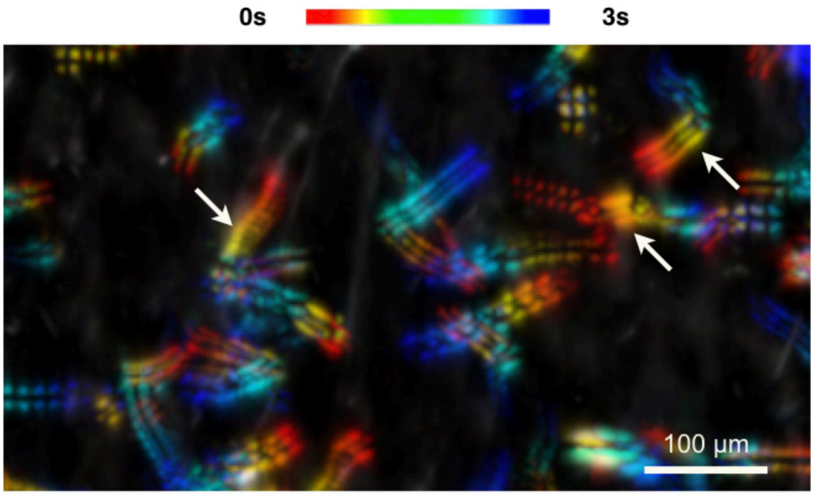

Supplementary Figure 1. Trajectories of solitons. At the time (indicated by white arrows), the first application of the electric field is removed, and the solitons instantaneously disappear. Then the second electric field is applied within about 5 s, the solitons appear at the same locations as where they disappeared.

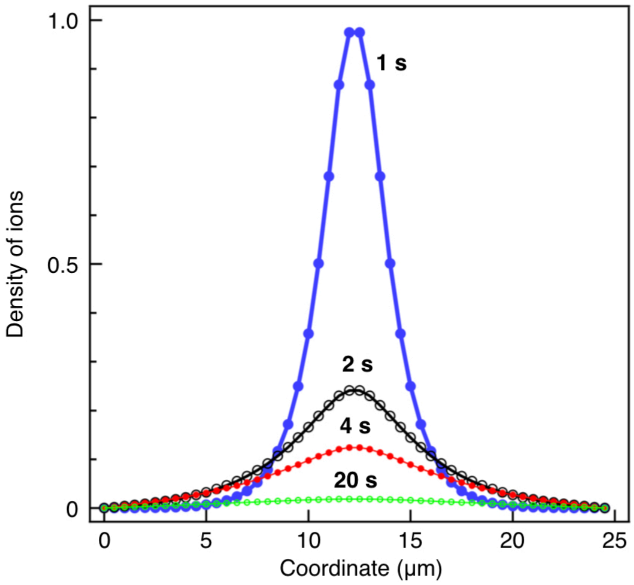

Supplementary Figure 2. Simulation results for the diffusion of ions in space. At an initial state at t = 0 s, the ions are assumed to be localised at 12.5 µm upon the generation of solitons. The curves of the spatial distribution of the ions at 1, 2, 4, and 20 s are given in blue, black, red and green, respectively.

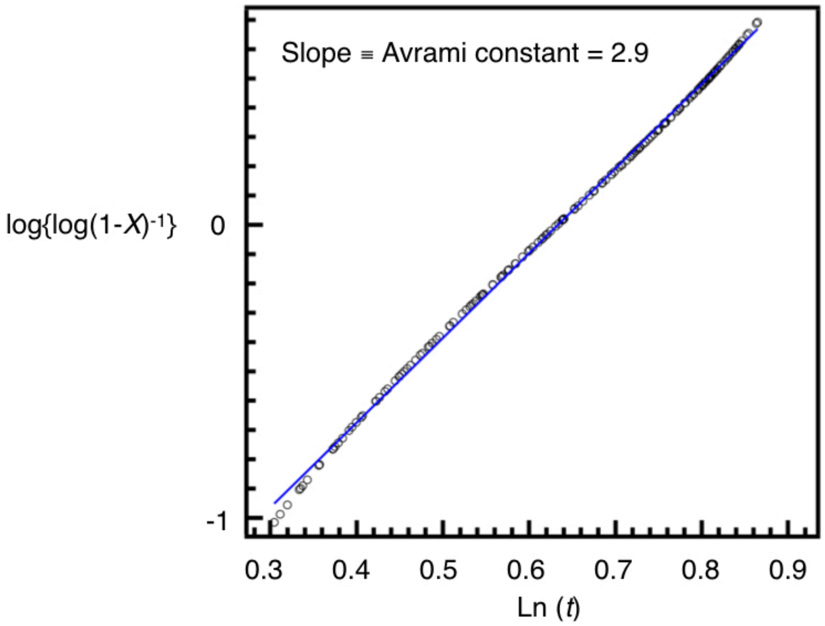

Supplementary Figure 3. Avrami analysis for soliton creation with the time-dependent soliton volume ratio, plotted on the Avrami scale.

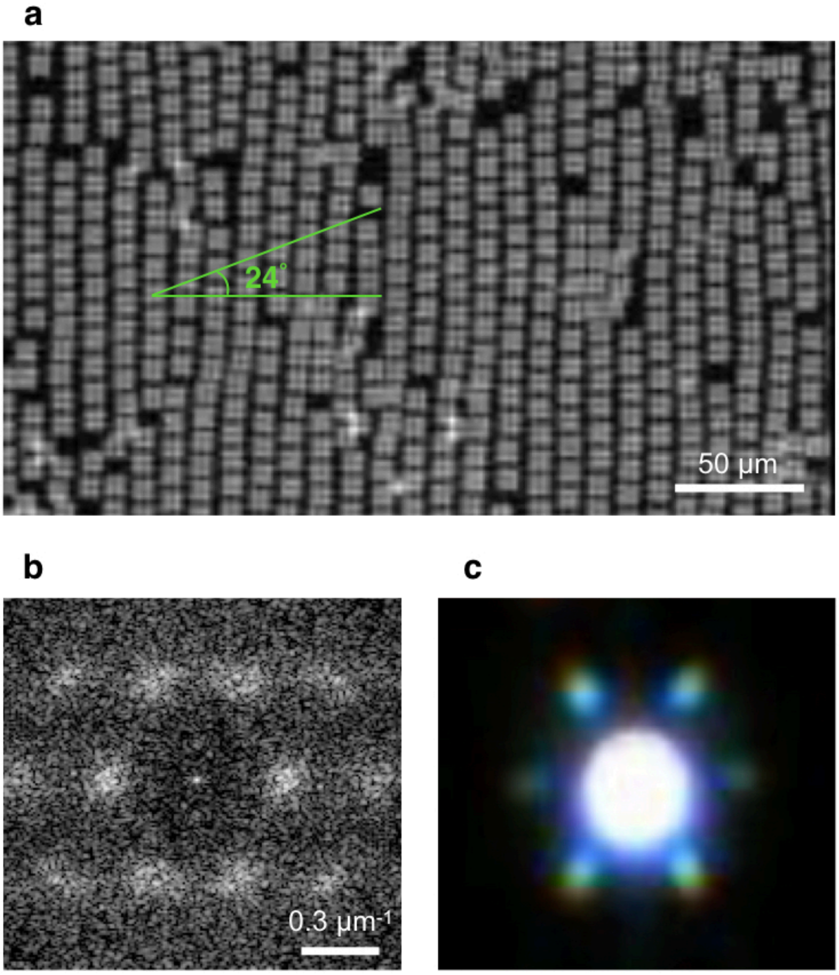

Supplementary Figure 4. (a) POM micrographs the centered rectangularly packed solitons. Scale bar, 50 μm. (b) Fast Fourier transform of (a). Scale bar, 0.3 μm^{-1}. The scale bar applies also to (c). (c) Conoscopy of (a).

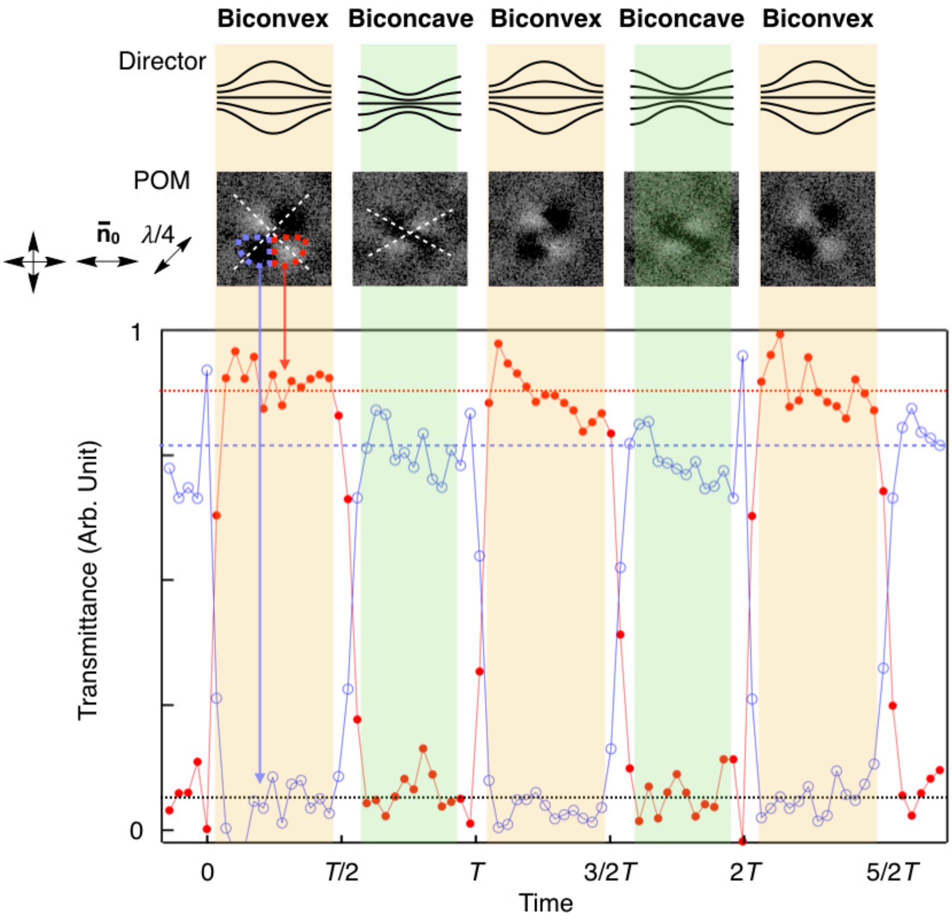

Supplementary Figure 5. Time evolution of the structural variation in the solitons observed with high-frame-rate polarising microscopy with a $\lambda/4$ plate. The periodicity of the applied electric field, T, is 0.1 s and the voltage is 6V.

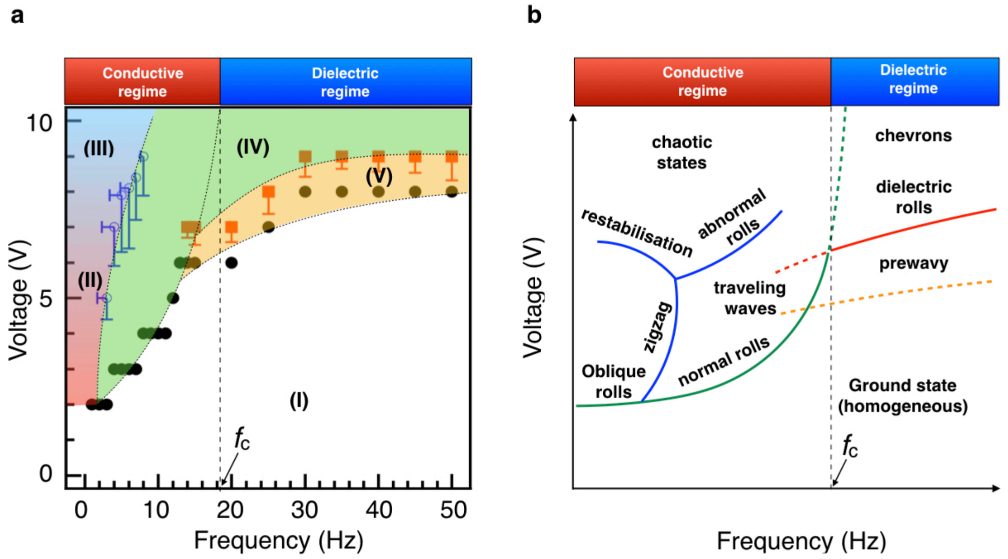

Supplementary Figure 6. Comparison state diagram of electrodynamic phenomena. (a) The state diagrams of the current soliton system. The hysteresis ranges by changing either the voltage or frequency are represented with error bars indicating the coexisting regions between the two distinct states. The lowest values correspond to the transition of the state by decreasing either the voltage or frequency. The points without bars have small hysteresis ranges less than 0.3 V in voltage or 0.8 Hz in frequency. (b) The typical conventional convective picture (For example, one can find a similar diagram in Supplementary Reference 5).

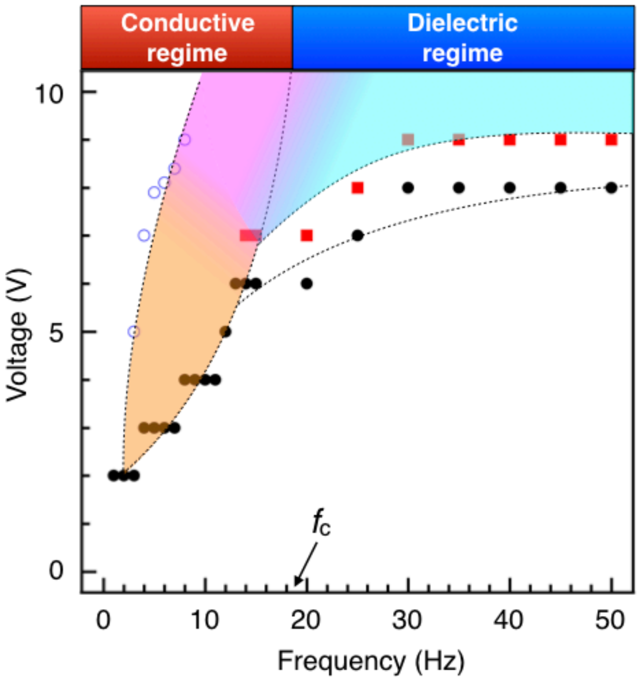

Supplementary Figure 7. State diagram showing different regimes in the soliton state. The areas with different colour coding correspond to different regimes of the dynamic solitons: orange for oscillation regime, purple and blue for swimming regime. In the purple or blue regimes, the solitons swim perpendicular or parallel to the alignment direction of the background molecules, $\overline{\mathbf{n}}_0$. In the shaded area between the purple-blue area, the motion of the solitons changes the directionality continuously as shown in Figs. 3c-h in the main text.

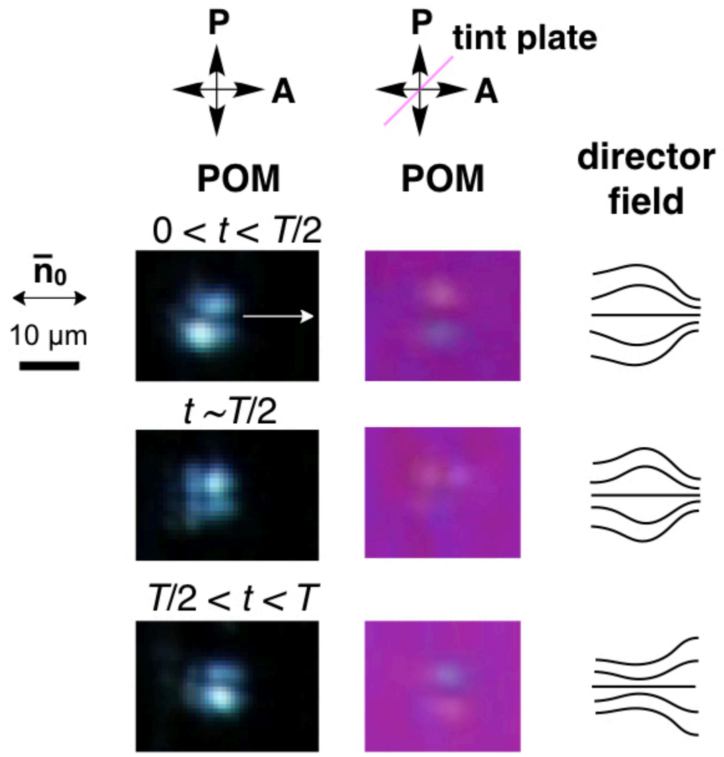

Supplementary Figure 8. Dynamic structure of the solitons during their swimming observed under POM. The white arrow in the upper picture shows the directionality of the motion. Scale bar, 10 µm.

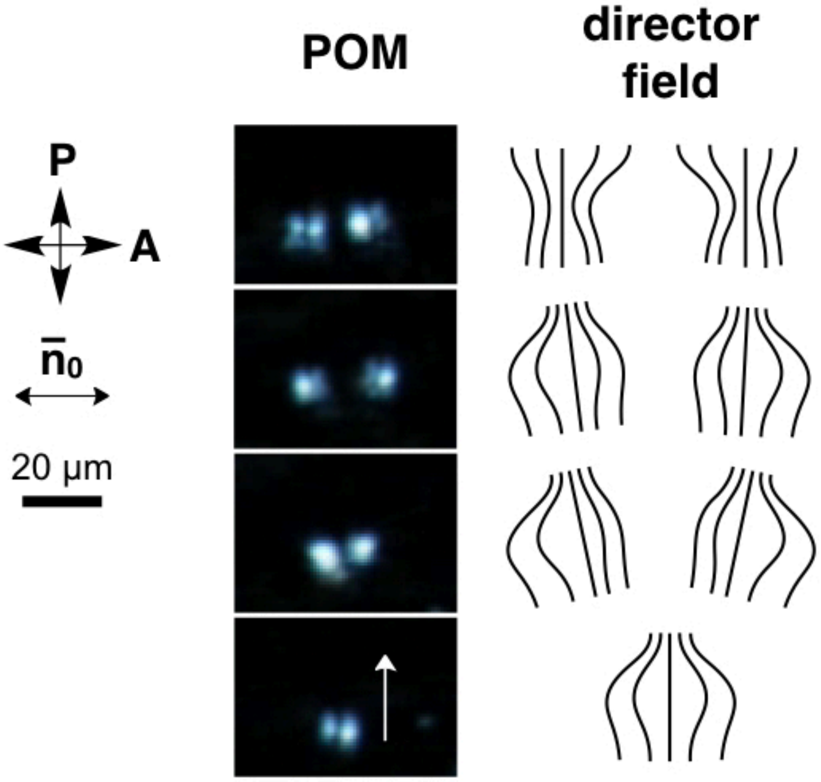

Supplementary Figure 9. Dynamic structure of the solitons upon their fractalization observed under POM. The white arrow in the bottom picture shows the directionality of the motion. Scale bar, 20 µm.

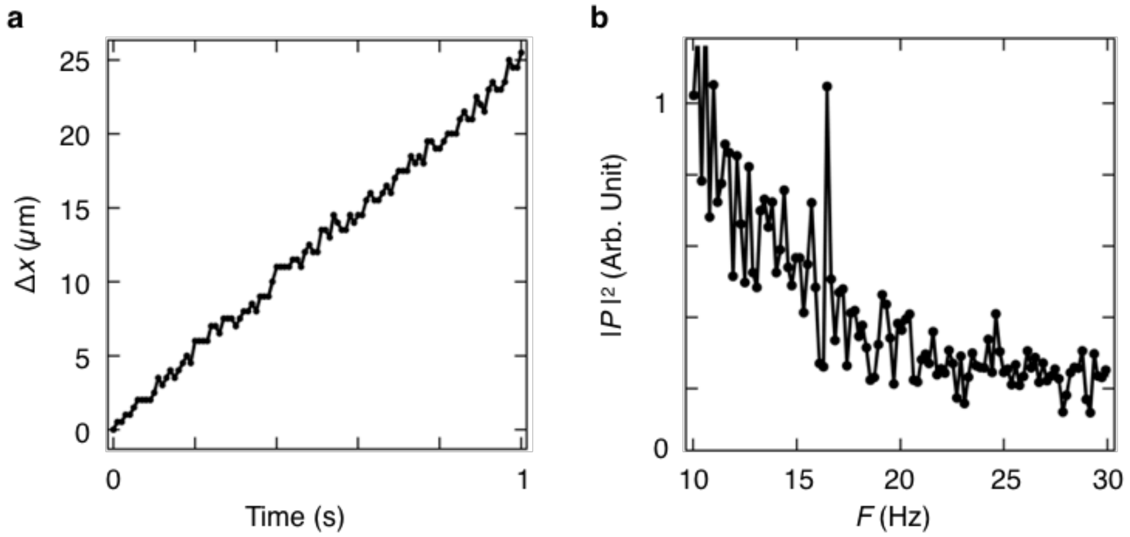

Supplementary Figure 10. Analysis of the go-and-stop motion of the solitons at an electric field of 16 Hz.

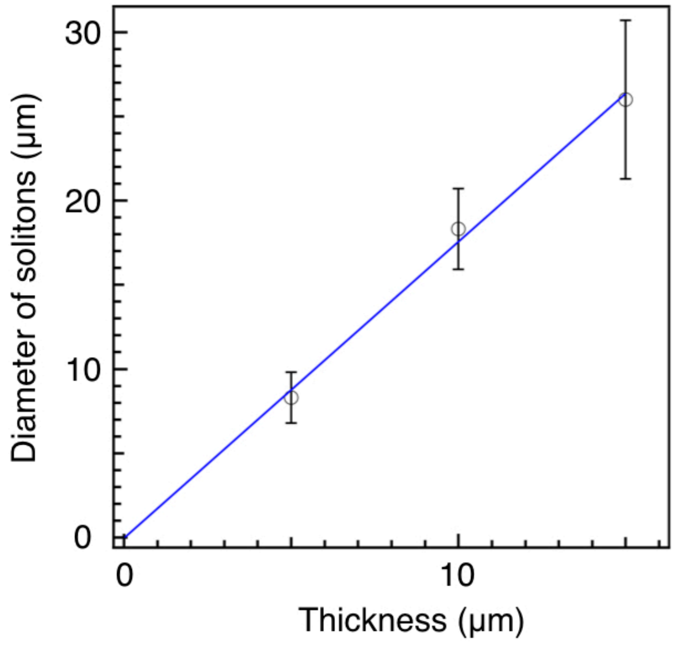

Supplementary Figure 11. Thickness dependence of the diameter of the solitons. The error bars correspond to the standard deviation deduced from more than ten data points for each thickness.

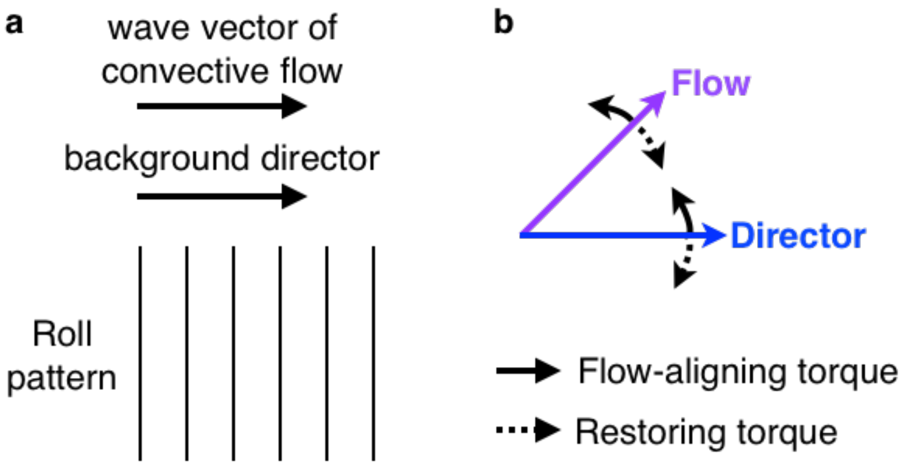

Supplementary Figure 12. (a) Schematic of the roll pattern in relation to the wave vector and the background director field. (b) Schematic of the feedback mechanism of oscillation of the director and flow field.

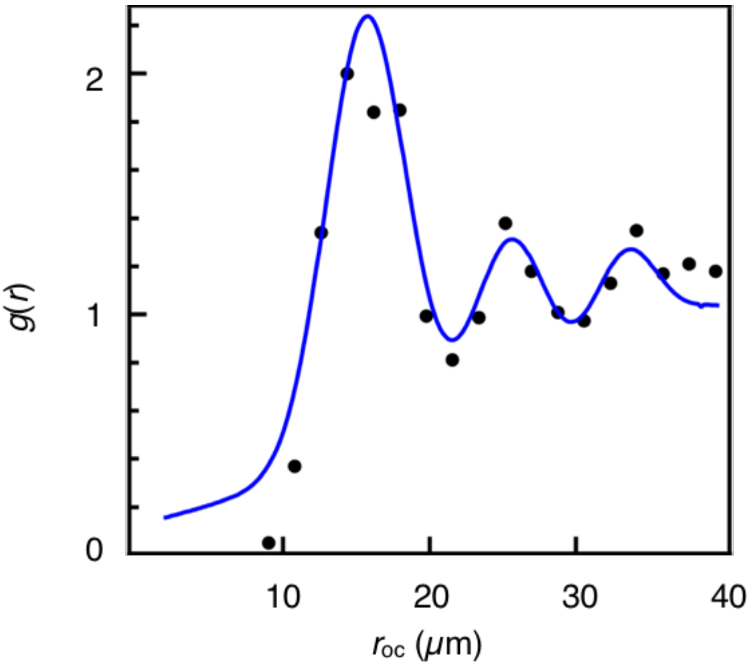

Supplementary Figure 13. Radial distribution function of centered rectangularly packed solitons.

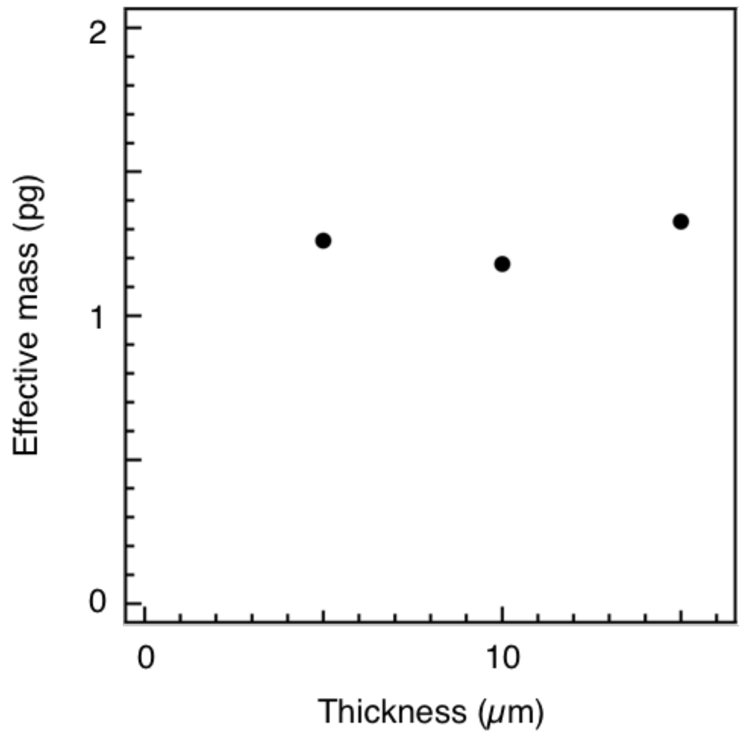

Supplementary Figure 14. Thickness dependence of the effective mass of the solitons.

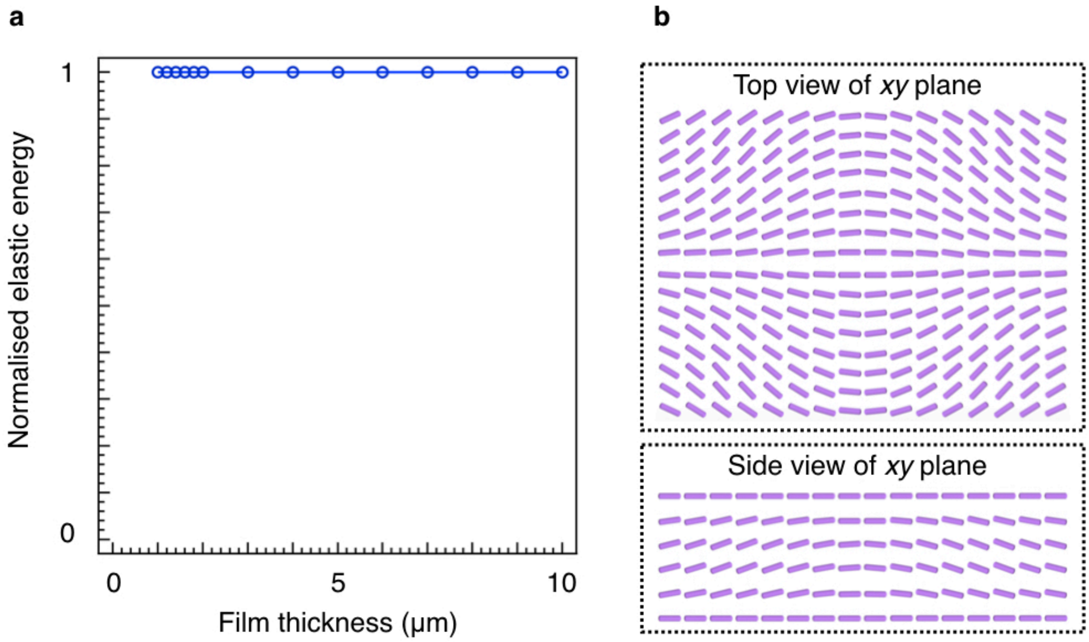

Supplementary Figure 15. (a) The elastic energy stored in an individual soliton as a function of the film thickness, calculated based on Eqs. 6 and 7 in the main text. The normalised elastic energy is the elastic energy divided by that of a 1 μm film. (b) The static director field obtained by the calculation.

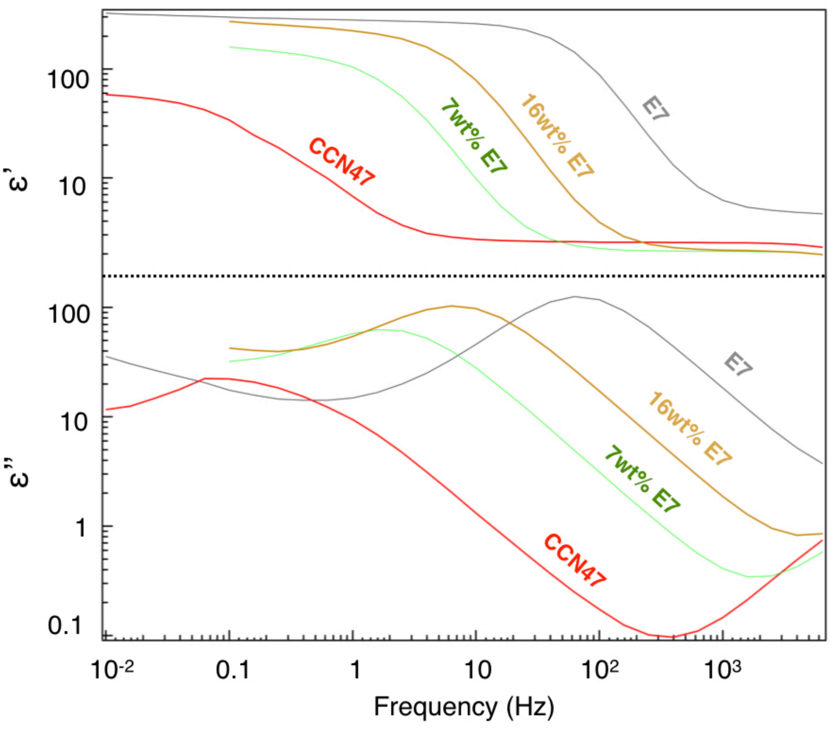

Supplementary Figure 16. Dielectric spectra of CCN47-E7 mixtures. Top panel: the real part of the complex dielectric constant as a function of the frequency. Bottom panel: the imaginary part of the complex dielectric constant as a function of the frequency.

Supplementary Information

Supplementary Note 1. Ion localisation in solitons

Ions play important roles in stabilising solitons. Here, we show an essential experimental result regarding ion localisation in solitons, i.e., the observation of the redistribution of ions. In the experiment, we first switch on an electric field to create solitons. At an arbitrary time, we remove the electric field, and the solitons instantly disappear. Then, we wait for Y seconds for the next application of the electric field to create solitons. If a localisation process of ions is involved in soliton generation, the diffusion or redistribution of ions should occur after the electric field is removed. We observe this by tracking the location at which the solitons are regenerated while tuning the waiting time Y. As a result, we find that solitons are regenerated at the same location as before the electric field is removed if Y is less than about 20 s (Supplementary Figure 1). Otherwise, they are randomly regenerated in space. This means that some ions are indeed accumulated at the center of the solitons upon their generation. Once the diffusion of ions completes after the electric field is removed, new solitons are randomly created in space at the second time of generation because there are new seeds for the solitons due to the localisation of ions. Taking a typical diffusion coefficient of the ions in liquid crystals of 2×10^{-9} m s^{-2}, it is calculated that the delocalisation of the ions takes about 20 s, which is consistent with the observed time scale (Supplementary Figure 2).

Supplementary Note 2. Analysis of the soliton growth process

A Kolmogorov–Johnson–Mehl–Avrami (KJMA) analysis of the structure-forming events provides information on how homogenously these events unfold and how their nucleation and growth processes proceed[1–4]. To estimate the occurrence frequencies of the electrically pumped solitons, we plotted the time evolution of the soliton volume ratio during their creation using the Avrami expression, $\log[\log[(1-X)^{-1}]] = m\log t + \log K$, where X, t, K, and m are the soliton volume fraction, time, temperature-dependent Avrami coefficient, and Avrami exponent, respectively (Supplementary Figure 3). We found that the overall creation process presents a good fit with the Avrami scale with an Avrami exponent of 2.9. Considering the random incidence of each soliton within space, as revealed by polarising microscopy, the creation of solitons

can be seen to be mediated by the homogeneous nucleation of soliton seeds, which is initiated by a localisation of ions, as discussed later, and the subsequent two-dimensional spreading of their network.

Now, we turn to consider why the KJMA model fits our data well. When creating solitons, switching on an electric field initiates the localisation of ions, and the localised ions becomes seeds that have potential to trigger the creation of the solitons. Let us assume the number of seeds at time 0 is N_0 and these can be activated to create solitons through a first-order-like reaction process. Then, the number inactivated seeds at time t can be written as $N_0 e^{-at}$, and the activation rate of the seeds to be nucleus of the solitons per unit time $aN_0 e^{-at}$. As a result, the emerging number of solitons is proportional to $N_0 - aN_0 e^{-at} \propto 1 - e^{-kt^n}$, which is in the same expression as that of the KJMA model and validates the usage of the model to evaluate the growth process of the solitons.

Supplementary Note 3. Packing of solitons

We analysed images of packed solitons to gain a better understanding of their packing states. Supplementary Figure 4a shows a swarm of solitons packed in a centered rectangular manner. A fast Fourier transform of Supplementary Figure 4a is shown in Supplementary Figure 4b, and the corresponding direct observation via the conoscopy, that is, the diffracted image of the packing state of the solitons, is shown in Supplementary Figure 4c. It shows clear consistency with the Fourier-transformed image.

Supplementary Note 4. Structure of solitons

As stated in the main manuscript, the solitonic structure is dynamic. When we observe the structure of the solitons under a POM by a conventional low-speed camera, which has a capturing rate lower than the oscillating frequency of the dynamic solitons, the solitons appear as a time-averaged structure. In this case, they look like bearing topological defects near their center. In order to clarify the time sequence of the structure, we perform high-frame-rate polarising microscopy with a $\lambda/4$ plate at 100 frames per second. In Supplementary Figure 5, the time evolutions of the transmittance in two

domains of the observed textures are plotted as a function of time along with a schematic director field and the textures at specified times. *T* is the periodicity of the applied electric field. Clearly, it is shown that the transmittance of the biconvex structure is stronger than that of the biconcave one. This suggests that the maximum deviation angle of the director in the biconvex structure is larger than that in the biconcave one. Another key feature is that the inclination angle of the brushes in the textures between the biconvex and biconcave structures is very different, indicated by the white dotted lines. Lastly, both the switching times from the biconvex to the biconcave and from the biconcave to the biconvex are of the order of several millisecond, and the switching time from the biconvex to the biconcave is shorter. These suggest the structural oscillation is elasticity-driven. All of these evidences confirm that the biconvex and biconcave structures are not a simple symmetric inversion of the director field, but the director field is more distorted in the biconvex structure.

Supplementary Note 5. Conditions of soliton creation - comparison between the current solitonic systems and conventional convective systems.

The state diagram (Figs. 2a-e in the main text, Supplementary Figures 6 and 7) infers an important connection between the current solitonic systems and conventional convective systems. We compare the known state diagram in a review by Eber et al.[5] (Supplementary Figure 6, right panel) with our state diagram (Supplementary Figure 6, left panel). It turns out that the G-, ||-, R- and ⊥-states appear in similar ranges with the ones in the conventional convective systems. The significant difference is that the Soliton state replaces the travelling waves and dielectric rolls regimes, and suppresses the chaotic regime.

Supplementary Note 6. Kinetics of solitons

In order to show the details of the kinetics of solitons, we show a state diagram in Supplementary Figure 7, focusing on the soliton state. The dynamic solitons just fluctuate around their equilibrium position at low frequencies (orange regime). At low

frequencies and high voltages (Purple regime), the solitons swim parallel to the direction of the background director. At higher frequencies and high voltages (Blue regime), the solitons swim perpendicular to the direction of the background director.

Since this regime is right next to the electrohydrodynamic regime ((V) $\perp$-state) where the direction of background flow is parallel to the direction of the background director, the swimming direction is dominantly determined by the flow as we stated in the Discussion part. In the narrow shaded region between the blue and the orange, the solitons change their swimming direction between parallel and perpendicular to the direction of the background director, as shown in Figs. 3c-i. In this regime, the background flow becomes slightly oblique, suggesting an additional travelling mode out of the *xz* plane becomes stronger.

Supplementary Note 7. Dynamic structure of solitons in motion

As stated in Supplementary Note 4, the high-frame-rate polarising microscopy is capable to capture dynamic properties of the structure of the solitons. In the swimming regime, we observe the structure of the solitons oscillates about an axis parallel to the direction of the solitonic motion as shown in Supplementary Figure 8.

Supplementary Note 8. Go-and-stop motion of swimming solitons

We observe the trajectory of the solitons upon their swimming, taken by a high-speed camera (Supplementary Movie 9). This allows us to analyse the accurate dynamics of the solitons. Supplementary Figure 10a demonstrates an increment of *x*-coordinate of a soliton as a function of time when the soliton is moving along *x* direction. It is clear that *x*-coordinate of the solitons averagely-increases, and oscillates. Supplementary Figure 10b shows a power spectrum of Supplementary Figure 10a, revealing the rate of the go-and-stop motion is about 16.5 Hz, consistent with the frequency of the electric field, 16 Hz. This fact suggests that the go-and-stop motion is caused by the switching between biconvex and biconcave structures upon solitonic swimming.

Supplementary Information

Supplementary Note 9. Size of solitons

The size of solitons is determined by the balance between the elastic energy and the surface anchoring. As discussed in the manuscript, the solitons carry out-of-plane deformation along the z axis. Therefore, it is expected that the size of the solitons is dependent on the sample thickness; that is, a thicker sample results in larger solitons. In Supplementary Figure 11, the linear dependence of the size of the solitons on the sample thickness is shown.

Supplementary Note 10. Feedback processes of the director and flow field through a flow-alignment process

The orientation of the director tends to be parallel to the flow field as a result of the flow-alignment property. Since the flow field deviates from the initial state after the reorientation of the director, the orientations of both the flow field and director oscillate as a result of a self-regulating procedure (Supplementary Figure 12).

Supplementary Note 11. Stabilisation of Solitons

Herein, we discuss why dynamic solitons have been so difficult to observe, despite the lengthy history of research on the electrohydrodynamics of liquid crystals, and, correspondingly, why our system is a special case. We again emphasise that the key property of the soliton system examined in this study is the frustration of dielectricity and conductivity. As shown in Fig. 2h of the main text, the Soliton-state appears only for $(-+)$ mixtures at moderately low conductivities in the range of $8 \times 10^{-9} < \sigma < 4 \times 10^{-8}\ \Omega^{-1}\mathrm{m}^{-1}$, which is two or three orders of magnitude lower than that of typical materials used in electrohydrodynamic studies but similar to previously reported values[6,7]. The importance of this middle conductivity region can be understood by considering the coupling between the space charge Q and the electric field E. In the lower-conductivity region (proportional to Q), the number of free ions is spatially limited; as a result, the viscous torque proportional to the time-averaged quantity[8], $\langle QE \rangle_T$, must destabilise the

ground planar state, but is too small to overcome the total stabilising force produced by surface anchoring and dielectric alignment in the framework of the Carr–Helfrich electrohydrodynamic effect. In contrast, in the high-conductivity regime in which Q is high and uniformly distributed in space, a viscous torque dominates and produces a turbulent flow. In the intermediate region, there is a sufficient number of ions for the Carr–Helfrich electrohydrodynamic effect to apply, but these can still be spatially localised. This consideration is well supported by the numerical calculation of electroconvective dynamics by Treiber and Kramer[9]. Overall, the value of $<QE>_{\mathrm{T}}$ effectively reflects the appearance of spatially localised waves corresponding to the Soliton-state.

Supplementary Note 12. Radial distribution of solitons in the centered rectangular packing state

The radial distribution function of the solitons in the centered rectangular lattice, $g(r_{\mathrm{cc}})$, was calculated using imaging analysis. The radial distribution function is calculated as the ratio of the average soliton density, $\rho(r_{\mathrm{cc}})$, at a distance of (r_{cc}) to the average density of solitons over the entire structure, ρ, as $g(r_{\mathrm{cc}}) = 2\rho(r_{\mathrm{cc}})\,\rho^{-1}$ (Supplementary Figure 13).

Supplementary Note 13. Relationship between the solitonic trajectories and the pair-wise potential curve

In Fig. 4 of the main text, it is shown that the solitonic dynamic behaviour upon a collision is changed depending on the positional offset, δ, of the solitons. Especially, when δ of two solitons in a collision event is about zero, i.e., a head-on collision, the solitons repel and make a vertical repulsion by a displacement of about 15 μm. This displacement is consistent with the value of the mean nearest-neighbor distance between solitons that is calculated by the pairwise potential curve (Fig. 6a of the main text). From this perspective, the collision is surely triggered by an elastic repulsion between the solitons.

Supplementary Note 14. Characterisation of the electrical and dielectric properties

The electric and dielectric responses of the liquid-crystal mixtures were characterised using dielectric spectroscopy (Solartron Analytical, impedance/gain-phase analyser 1260A with dielectric interface system 1296A). We focused on the low-frequency range (0.01–10^4 Hz), in which the main response originates from the charge polarisation of the mobile ions[10]. The mixtures were confined between parallel glass plates with thin layers of indium tin oxide and were subjected to a 0.05 V AC electric field sweeping a frequency range of 0.01–10^4 Hz. Supplementary Figure 16 shows the dielectric spectra produced at various weight ratios of E7 to the host CCN47. Clearly, an increase in the ratio of E7 increases both the ionic relaxation frequency and dielectric strength as a result of the increased number of movable ions. Using the ionic characterisation method introduced by Sawada et al.[10], we calculated the conductivities of the mixtures, as shown in Fig. 2h of the main text.

Supplementary Information

Supplementary References

[1] Avrami, M. Kinetics of phase change. I. General theory. *J. Chem. Phys.* **7**, 1103–1112 (1939).

[2] Avrami, M. Kinetics of phase change. II. Transformation-time relations for random distribution of nuclei. *J. Chem. Phys.* **8**, 212–224 (1940).

[3] Avrami, M. Kinetics of phase change III. Granulation, phase change, and microstructure. *J. Chem. Phys.* **9**, 177–184 (1941).

[4] Torrens-Serra, J., Venkataraman, S., Stoica, M., Kuehn, U., Roth, S. & Eckert, J. Non-isothermal kinetic analysis of the crystallization of metallic glasses using the master curve method. *Materials* **4**, 2231–2243 (2014).

[5] Éber, N., Salamon, P. & Buka, Á. Electrically induced patterns in nematics and how to avoid them. *Liq. Cry. Rev.* 4, 101–134 (2016).

[6] Brand, H. R., Fradin, C., Finn, P. L., Pesch, W. & Cladis, P. E. Electroconvection in nematic liquid crystals: Comparison between experimental results and the hydrodynamic model. *Phys. Lett. A.* **235**, 508–514 (1997).

[7] Kramer, L. & Pesch, W. Convection instabilities in nematic liquid crystals. *Ann. Rev. Fluid. Mech.* **27**, 515–539 (1995).

[8] Treiber, M. & Kramer, L. Coupled complex Ginzburg–Landau equations for the weak electrolyte model of electroconvection. *Phys. Rev. E* **58**, 1973–1982 (1998).

[9] Sawada, A., Nakazono, Y., Tarumi, K. & Naemura, S. Complex dielectric constant of liquid crystal materials containing ionic impurities in low frequency region. *Mol. Cryst. Liq. Cryst.* **318**, 225–242 (1998); Sawada, A., Nakazono, Y., Tarumi, K. & Naemura, S. Space charge polarization by ions in a liquid crystal material. *Mol. Cryst. Liq. Cryst.* **331**, 457–464 (1999); Sawada, A., Tarumi, K. & Naemura, S. Novel Characterization method of ions in liquid crystal materials by complex dielectric constant measurements. *Jpn. J. Appl. Phys.* **38**, 1423–1427 (1999).
