# Supplementary material for: Kinetics of motile solitons in nematic liquid crystals
Source: Nat Commun. 2020 Jun 26;11:3248. doi: 10.1038/s41467-020-16864-8 (PMC7319993; doi:10.1038/s41467-020-16864-8)
Supplement: Supplementary file 2 — Description of Supplementary Files [file 41467_2020_16864_MOESM2_ESM.docx]

**Description of Additional Supplementary Files**

**File Name: Supplementary Movie 1**

**Description:** Time-dependent creation process of a soliton network. Real-time movie captured through the crossed polarisers of a polarising microscope showing soliton nucleation at random locations. The applied voltage is 10 V at 12 Hz, and $\overline{\mathbf{n}}$_0_ points horizontally.

**File Name: Supplementary Movie 2**

**Description:** Time-dependent oscillatory process of each soliton. Real-time movie captured with the bright field illumination of disclination loops elongated perpendicular to $\overline{\mathbf{n}}$_0_ and oscillating parallel to $\overline{\mathbf{n}}$**_0_**. The loop oscillation phases are evenly split, with half oscillating in phase with the electric field and the remainder oscillating 180° out of phase. The applied voltage is 10 V at 20 Hz. $\overline{\mathbf{n}}$_0_ points horizontally.

**File Name: Supplementary Movie 3**

**Description:** Soliton dynamics. Real-time movie captured through the crossed polarisers of a polarising microscope of soliton motions tracked at 8.3 V and 20 Hz. $\overline{\mathbf{n}}$_0_ points horizontally.

**File Name: Supplementary Movie 4**

**Description:** **Soliton dynamics.** Real-time movie captured with the crossed polarisers of a polarising microscope of soliton motion tracked at 8.5 V and 20 Hz. $\overline{\mathbf{n}}$_0_ points horizontally.

**File Name: Supplementary Movie 5**

**Description:** Real-time movie captured through the crossed polarisers of a polarising microscope of soliton motion tracked at 8.8 V and 20 Hz. $\overline{\mathbf{n}}$_0_ points horizontally.

**File Name: Supplementary Movie 6**

**Description:** Real-time movie captured through the crossed polarisers of a polarising microscope of soliton motions tracked at 8.9 V and 20 Hz. $\overline{\mathbf{n}}$_0_ points horizontally.

**File Name: Supplementary Movie 7**

**Description:** Soliton dynamics. Real-time movie captured through the crossed polarisers of a polarising microscope of soliton motions tracked at 9.3 V and 20 Hz. $\overline{\mathbf{n}}$_0_ points horizontally.

**File Name: Supplementary Movie 8**

**Description:** Soliton dynamics. Real-time movie captured through the crossed polarisers of a polarising microscope of soliton motions tracked at 9.6 V and 20 Hz. $\overline{\mathbf{n}}$_0_ points horizontally.

**File Name: Supplementary Movie 9**

**Description:** Kinetics of solitons at 8 V and 16 Hz captured at 100 fps.

**File Name: Supplementary Movie 10**

**Description:** Collisions of solitons.

**File Name: Supplementary Movie 11**

**Description:** Binarised real-time movie captured through the crossed polarisers of a polarising microscope of the fractalised proliferation of soliton trajectories tracked at 10 V and 20 Hz. The background of the movie is averaged over time such that the soliton shapes appear sharp. $\overline{\mathbf{n}}$_0_ points horizontally.

**File Name: Supplementary Movie 12**

**Description:** Real-time movie captured through the crossed polarisers of a polarising microscope of the coexistence of oblique rolls and solitons. The voltage is 8 V with the frequency continuously down-swept from 25 to 20 Hz. $\overline{\mathbf{n}}$_0_ points horizontally.
